# A Rare Presentation of Orbital Castleman's Disease

**DOI:** 10.1155/2020/1012759

**Published:** 2020-01-03

**Authors:** Ruchi Goel, Akash Raut, Ayushi Agarwal, Shweta Raghav, Sumit Kumar, Simmy Chaudhary, Priyanka Golhait, Sushil Kumar, Ravindra Saran

**Affiliations:** ^1^Guru Nanak Eye Centre, Maulana Azad Medical College, New Delhi 110002, India; ^2^Kallam Anji Reddy Campus, L V Prasad Marg, Banjara Hills, Hyderabad 500034, India; ^3^Department of Pathology, Govind Ballabh Pant Post Graduate Institute of Education and Medical Research, New Delhi 110002, India

## Abstract

Castleman's disease (CD) is an uncommon group of atypical lymphoproliferative disorders. Extranodal involvement such as the orbit is extremely rare. We aim to report a case of a 62-year-old male who presented with left painless proptosis for the past three years. Examination revealed a firm, lobulated mass in the left superotemporal orbit, displacing the globe inferomedially. A well-defined extraconal orbital lesion encasing the left lateral rectus muscle with intraconal extension was seen on Magnetic Resonance Imaging (MRI) that led to the provisional diagnosis of left solitary encapsulated venous malformation. Excision of the mass via lateral orbitotomy was performed. However, on histopathology, the features were consistent with a mixed-cell variant of Castleman's disease. A detailed systemic workup was unremarkable. Proptosis resolved after surgery and no recurrence was noted in the three-year follow-up. To the best of our knowledge, this is the first case report of a mixed-cell variant of unicentric orbital CD without any systemic features. This case highlights the importance of including CD in the differential diagnosis of well-defined orbital lesions so as to enable its early detection and timely management.

## 1. Introduction

Castleman's disease (CD), a rare entity first described in 1956 by Dr. Benjamin Castleman, is a group of atypical lymphoproliferative disorders [1, 2]. Also called as angiofollicular lymphoid hyperplasia, it can present as either a unicentric (one site involvement) or a multicentric disease (more than one site involvement) [3]. On the basis of histopathology, four subtypes are found, namely, the hyaline-vascular variant, plasma cell variant, mixed-cell type, and plasmablastic type (plasmablasts expressing Human Herpes Virus- (HHV-) 8 antigen) [4]. Extranodal involvement such as the orbit is extremely uncommon, and so far, very few cases of orbital CD have been reported in the literature [5]. The most common histopathological type of orbital CD is the hyaline-vascular variant (approximately 90% of cases) which is usually unicentric, clinically presenting as a gradually progressive orbital mass. We describe a rare case of unicentric mixed-cell variant orbital CD that was initially misdiagnosed as a solitary encapsulated venous malformation.

## 2. Case

**Figure 1:**
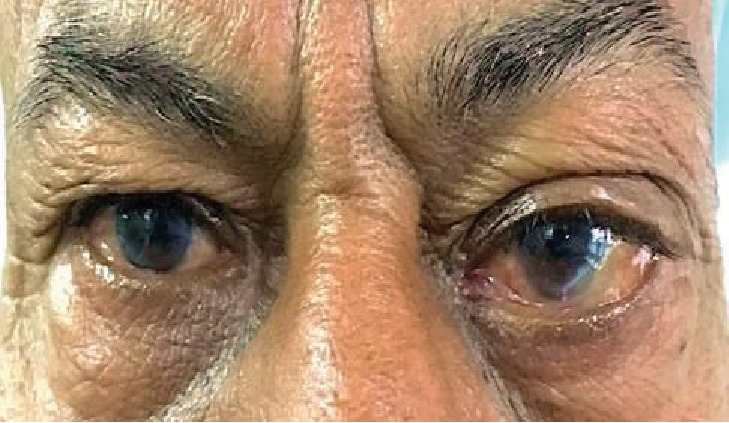
Clinical photograph showing left superior orbital fullness displacing the eyeball inferomedially.

A 62-year-old man presented with protrusion of the left eye for the past three years. It was insidious in onset, painless, and gradually progressive. There was no history of trauma, systemic illness, previous surgery, or diplopia. The best corrected visual acuity (BCVA) in the right eye was 20/200 and in the left eye 20/50. On examination, a nontender, nonpulsatile, lobulated, firm mass was palpated in the left superotemporal region, not associated with a change in size on valsalva. A horizontal dystopia of 2 mm and a vertical dystopia of 4 mm were observed displacing the eyeball inferomedially (Figure 1). The extraocular movement of the left eye was limited in levoelevation. Pupillary reaction and fundoscopy were unremarkable. No associated ocular involvement was detected. The systemic examination was within normal limits with no lymphadenopathy or organomegaly. A complete blood workup, including immunological profile, showed a relatively increased Erythrocytic Sedimentation Rate (ESR) of 25 mm/1^st^ hour. The rest of the parameters were within normal limits. Immunocompromised status was ruled out. The ultrasound B-scan of the left orbit revealed a mass lesion of low internal reflectivity in the lacrimal gland region.

**Figure 2:**
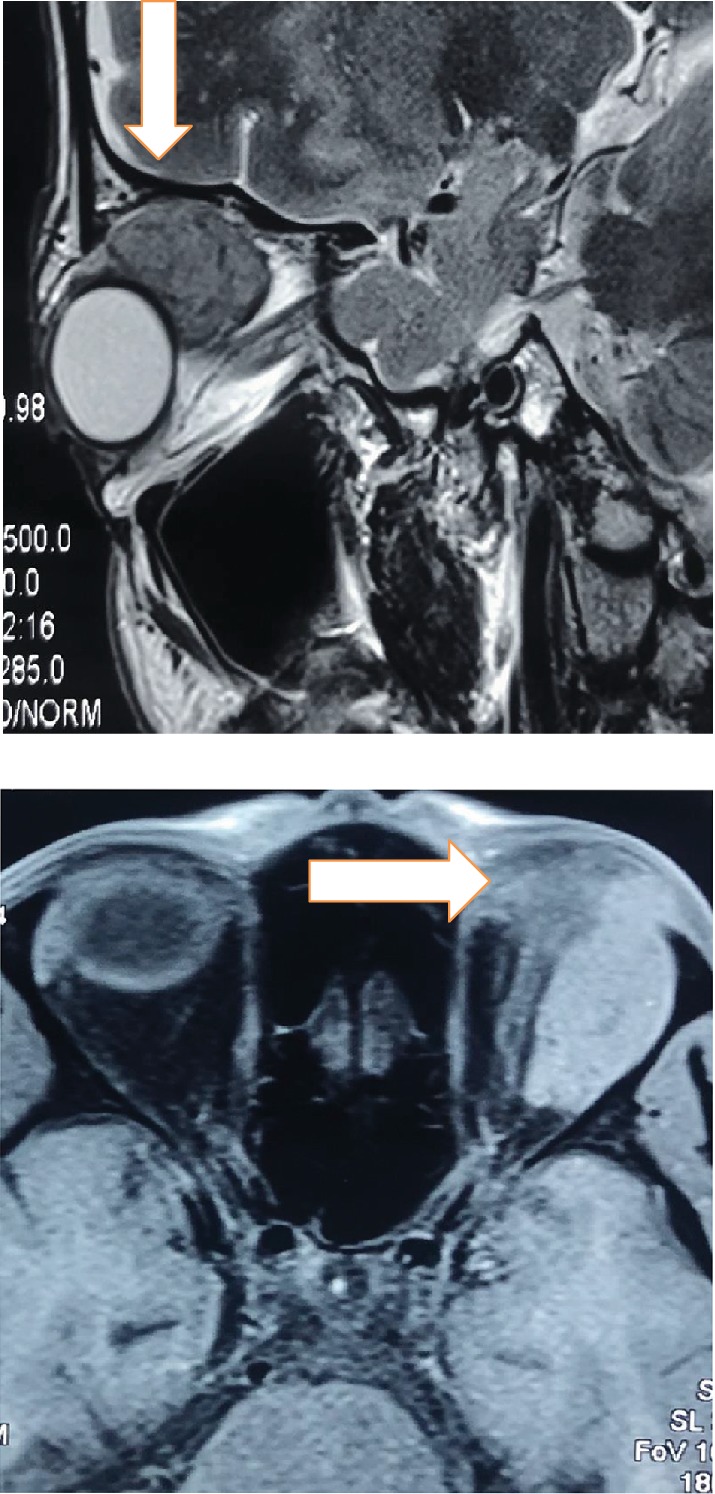
CE-MRI (Contrast-Enhanced Magnetic Resonance Imaging) showing left well-defined orbital lesion in the extraconal compartment abutting the left lateral rectus muscle with intraconal extension.

A Contrast-Enhanced Magnetic Resonance Imaging (CE-MRI) of the orbit revealed a well-defined soft tissue lesion predominantly in the superolateral aspect of the extraconal compartment of the left orbit with intraconal extension, abutting the left lateral rectus muscle (Figure 2). The lesion measured 3.2 *cm* (*anteroposterior*) × 1.6 *cm* (*transverse*) × 2.4 *cm* (*craniocaudal*). It appeared isointense on T1 and hyperintense on T2-Weighted Image (T2WI) with tiny hypointense foci on T1 as well as T2WI. Dynamic contrast-enhanced scans revealed progressive accumulation of contrast with persistence on delayed sequences with the lesion showing diffuse restriction. The lesion reached up to the lateral orbital wall laterally and the roof of the orbit superiorly. Since a provisional diagnosis of left solitary encapsulated venous malformation with bilateral immature senile cataract (*right* > *left*) was made, the patient was planned for mass excision via lateral orbitotomy.

**Figure 3:**
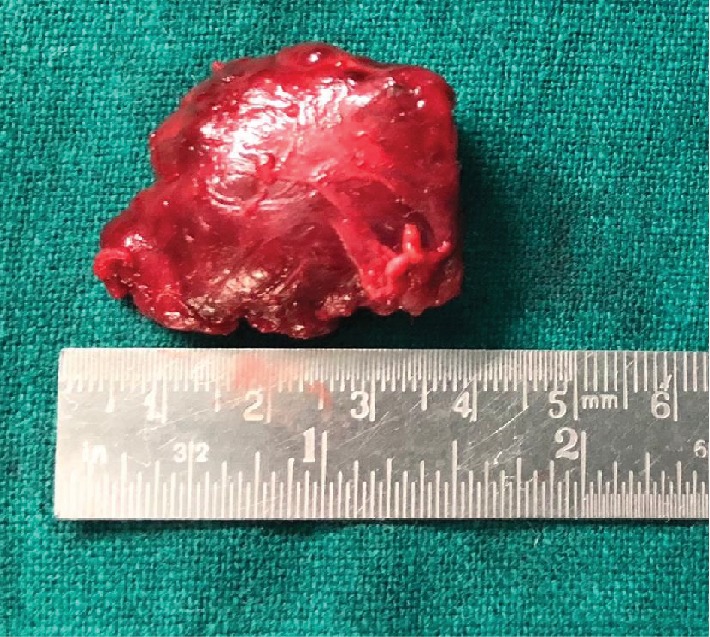
Gross examination showing a 3.5 × 3 × 2 cm well-defined, greyish-red, lobulated, and firm mass.

**Figure 4:**
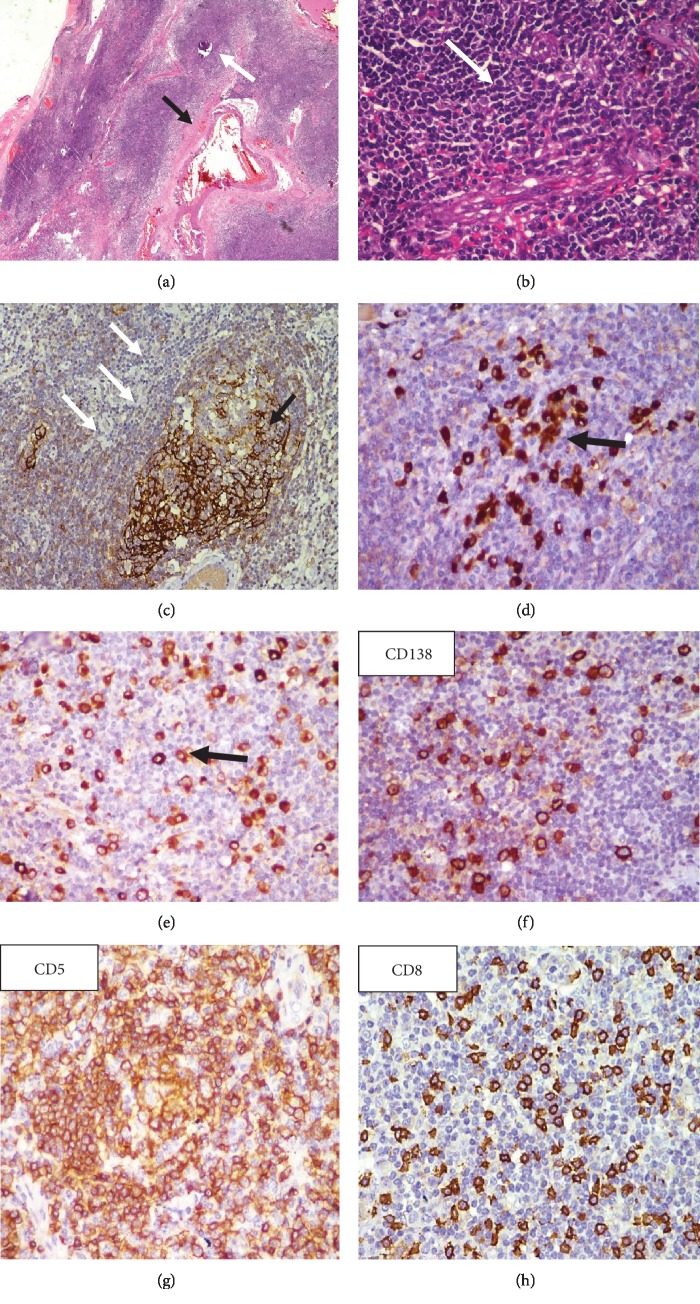
Histopathology suggestive of extranodal CD revealed (a) extranodal tissue showing a dilated blood vessel (black arrow) with multiple sheaths of lymphoid cells with attempted ill-formed follicles (white arrow) (HES2X), (b) mature looking lymphocytes (white arrow) (HES40X), (c) follicular dendritic cells positive for CD23 (black arrow) with garlanding (“onion skinning”) of lymphocytes around it (white arrow), (d) polyclonal expression of kappa and (e) lambda, and (f) CD138-positive for plasma cells (g) CD5-positive T cells and (h) cytotoxic CD8 cells.

Macroscopically, a 3.5 × 3 × 2 *cm* well-defined, greyish-red lobulated, firm mass was excised (Figure 3). Histopathological examination revealed sheets of mature-looking lymphoid tissue with attempted pseudofollicle formation and interfollicular hyalinized thick-walled blood vessels. Focal eccentric layering of the mantle zone (“onion skinning”) was present. There was increased reticulin framework in the interfollicular area. On immunohistochemistry, an intermixed population of CD5 and CD20 with numerous plasma cells highlighted by CD138 was noted. Polyclonal expression of both kappa and lambda was seen (Figure 4). These findings were consistent with extranodal Castleman's disease of mixed-cell variant. Systemic involvement was ruled out as serum Interleukin-6 levels, gamma globulins, and Computed Tomography (CT) of the chest, abdomen, brain, and pelvis were unremarkable. A confirmatory diagnosis of unicentric, mixed-cell type of orbital CD was made. Surgical excision resulted in complete resolution of proptosis.

## 3. Discussion

Orbital CD, an extremely rare entity, most commonly presents as a progressive painless swelling resulting in proptosis or as B symptoms, i.e., weight loss, fever, and night sweats in presence of systemic involvement. The aetiology is multifactorial. Viral, neoplastic, and inflammatory mechanisms play an important role in the pathogenesis of unicentric CD (UCD). Of all these, the primary driving force is believed to be Neoplastic Follicular Dendritic Cells (FDCs). Aberrant production of interleukin-6 (IL-6) has also been implicated in unicentric CD [6]. In immunocompromised conditions, HHV-8 (Human Herpes Virus/Kaposi-sarcoma virus), a lymphotropic virus, escapes the host immune response and replicates in the tissues, resulting in uncontrolled cytokine production mainly seen in multicentric CD [6].

Associated posterior segment or intracranial involvement must always be ruled out [2]. CD-like clinicopathological findings can be seen in lymphomas, Ig-G4-related disease, autoimmune disorders (rheumatoid arthritis, systemic lupus erythematosus), Idiopathic Orbital Inflammatory Disease (IOID), and metastatic lesions. Histopathological examination with IHC remains the gold standard for confirmatory diagnosis. Various management options are available depending upon the location (Table 1). A complete surgical excision in this case was curative, with no signs of recurrence seen during the follow-up of three years.

To conclude, CD must be included as a differential diagnosis of a well-defined orbital lesion. To the best of our knowledge, this is the first case report of a unicentric, mixed-cell variant of orbital CD in the absence of systemic features. A step-wise multidisciplinary approach is crucial for an early diagnosis and appropriate treatment.

## Figures and Tables

**Table 1:** Management of CD: an overview.

	Treatment	Prognosis
Unicentric (orbital) CD	Surgery (cornerstone)±neoadjuvant chemotherapy alternative: radiotherapy (i) If associated ocular involvement—trial of steroids (unless contraindicated) followed by radiotherapy	Surgery: excellent prognosis with 10-year survival rate > 95% Radiotherapy alone: 2- year survival rate—approximately 80% [7]

Multicentric CD	(i) Cytotoxic chemotherapy (CHOP/etoposide/CVAD/COP) (ii) Monoclonal antibody—rituximab (+CHOP) (iii) Corticosteroids (limited role) (iv) Emergent therapies: IL-6 receptor antagonist (tocilizumab, siltuximab), anakinra (IL-1 receptor antagonist), and autologous stem cell transplantation (v) Antiviral agents (in combination/maintenance therapy)	Multicentric CD has a long-term poor prognosis (worst in idiopathic MCD) Emergent therapies including rituximab show a better survival rate, but larger multicenter studies are required

CHOP: cyclophosphamide, doxorubicin, vincristine, and prednisolone; CVAD: cyclophosphamide, vincristine, doxorubicin, dexamethasone; COP: cyclophosphamide, vincristine, prednisolone.
